# The Historical Case for a Strong and Diverse Neurology Clerkship Leadership Team

**DOI:** 10.1212/NE9.0000000000200149

**Published:** 2024-11-06

**Authors:** Ashley Paul, Doris G. Leung, Carlos G. Romo, Vinay Chaudhry, Justin C. Mcarthur, Eric H. Kossoff, Jessica Nance, Charlene E. Gamaldo, Rachel Marie E. Salas

**Affiliations:** From the Department of Neurology (A.P., D.G.L., C.G.R., J.C.M., E.H.K., J.N., C.E.G., R.M.E.S.), School of Medicine, Johns Hopkins University, Baltimore, MD; and Division of Neuromuscular Disorders (V.C.), School of Medicine, University of North Carolina, Chapel Hill.

## Abstract

The role of the clerkship director has evolved significantly over the past century and now requires a diverse range of skills to meet the rigorous standards set by national accrediting bodies such as the Liaison Committee on Medical Education. We conducted a historical exploration, spanning the past 43 years, of the educational practices in the Neurology Department at Johns Hopkins University School of Medicine. We learned that no entity is responsible for documenting the history of the clerkship. Three distinct areas of focus represent the essential pillars of our clerkship: (1) building a diverse, equitable, and inclusive leadership team with complementary skill sets; (2) establishing medical education as a career path with institutional support and promotion; and (3) planning and supporting the transition of clerkship roles. These pillars facilitate an academic environment that promotes professional well-being and work-life integration, the development of opportunities for educational scholarship and professional development, and the identification, recruitment, and training of future medical educator leaders. This historical review underscores the importance of implementing a structured approach to organizing clerkships. Structure would facilitate innovation and contextual paradigm shifts in adult learning, shaping progress for the future. Furthermore, institutions should document the biography of the clerkship and neurology education. A biography would help maintain compliance with accrediting bodies, inform future planning based on outcomes of decisions made by past leaders, maintain continuity in the long-term vision of the neurology clerkship, ensure smooth transitions in leadership, and preserve institutional memory and legacy.

## Introduction

The formation of the Neurology Core Clerkship (NCC) at Johns Hopkins School of Medicine (JHSOM) and the evolution of its leadership provide context for examining today's evolving educational paradigm. The need for evidence-based education standards originated more than a century ago.^1^ During the 20th and 21st centuries, consortiums such as the Alliance for Clerkship Education have helped to disseminate standards for clerkship education and clerkship director (CD) competencies that include the ability to integrate scientific rigor, problem-based education, core professional competencies, and health systems science (HSS) in addition to basic and clinical sciences.^1^ In response to rising national standards for the professional development of leaders in medical education, JHSOM established the current clerkship structure, specifically the core neurology clerkship, which has transformed from a single directorship to a directorial team.

## National Trends in Clerkship Education

### The Evolving National Definition of CD Responsibilities

**Figure 1:**
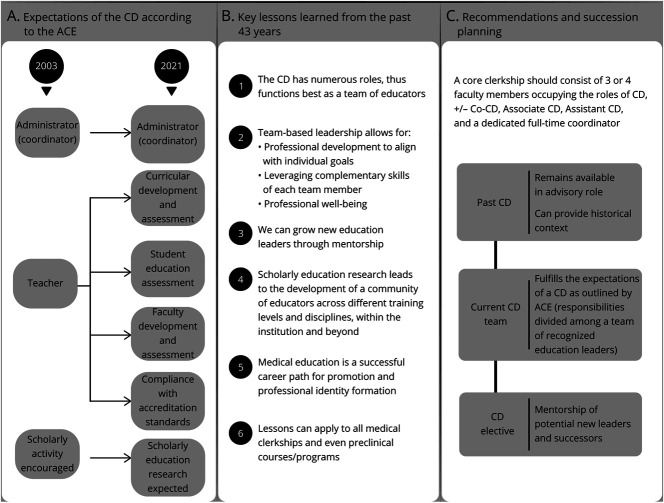
Evolving Expectations, Key Lessons Learned, and Recommendations (A) Expectations of the CD according to the ACE. (B) Key lessons learned from the past 43 years. (C) Recommendations and succession planning. ACE = Alliance for Clinical Education; CD = clerkship director.

In 2003, the Alliance for Clinical Education (ACE) published a guideline outlining the expectations for a core CD.^2^ At that time, the CD had 3 primary roles: administrator, teacher, and scholar (Figure 1A). CDs oversaw administrative tasks (e.g., managing rotation sites, scheduling, and budgeting). CDs were also expected to serve as effective teachers well-versed in curricular design, instruction, evaluation, assessment, and feedback. Professional development, innovation, and scholarship were recognized as crucial for career development. ACE recommended choosing experienced clinicians as CDs, which reflected the assumption that an experienced clinician was also an effective educator. Although this may be true, an evidence-based association has never been established.

In 2021, ACE recognized the CD's evolving role and published updated guidelines (Figure 1A).^1^ In addition to administrative responsibilities and curricular development, CDs must handle faculty recruitment, development, evaluation, and feedback and demonstrate effective mentorship skills, interpersonal skills, and adaptability.^1^ The guidelines emphasized the need for diverse, equitable, and inclusive clerkships, reflecting the changing demographics of medical schools and the US population and a national survey of neurology CDs.^3,4^ The Liaison Committee on Medical Education now centrally monitors clerkships to meet school objectives and curricular standards. Hence, CDs must possess the aptitude to meet accreditation criteria.^5^

### The Clerkship's Role in Integrating HSS Into the Curriculum

The ACE guidelines recommend the integration of HSS concepts into the clerkship curriculum. In addition to basic and clinical sciences, HSS is the third pillar of medicine. HSS encompasses a holistic array of complex factors (e.g., social determinants of health, high-value care, health care structure, and informatics) that affect patients and populations.^6^ Many institutions are now expected to have a longitudinal HSS curriculum. The ACE guidelines urge CDs to consider how students can recognize important HSS concepts in day-to-day clinical experiences. It is critical to professional and leadership development for students to think beyond disease and pathology.

### National Guidelines for Full-Time Equivalent Employees

ACE established guidelines for salary support and administrative resources that should be allocated to clerkships: a full-time equivalent (FTE) for the CD at 50% (25% for administration and 25% for curriculum development and scholarly activities) and a full-time administrative assistant.^2^ In 2005, one-third of neurology CDs reported receiving less than 5% FTE.^1^ There has been a national trend toward increasing support for neurology CDs: a 2017 national survey indicated that the mean FTE for neurology CDs was 24%, with one-third of respondents receiving 26%–50% FTE.^4^ Furthermore, approximately 57% of clerkships have an assistant, associate, or codirector with protected time (mean FTE 17%).^4^ The average support for coordinators has increased to 47% FTE.^4^ An updated survey was completed in 2022, the results of which have not yet been published.

## Merging Hopkins Tradition With National Medical Education Standards and Guidelines

Sir William Osler, who published more than 200 articles in neurology and completed 800 autopsies of the nervous system, is among those credited with popularizing the clerkship as a model for teaching students to observe and treat clinical patients.^7^ The model's origin can be traced to various sources, including a similar apprenticeship system at the New Orleans School of Medicine that predates the Civil War.^8^ In Osler's 1901 publication on teaching medicine, he emphasized the significance of clinical neurology, referencing a twice-weekly rotation to learn about nervous system diseases.^9^

**Figure 2:**
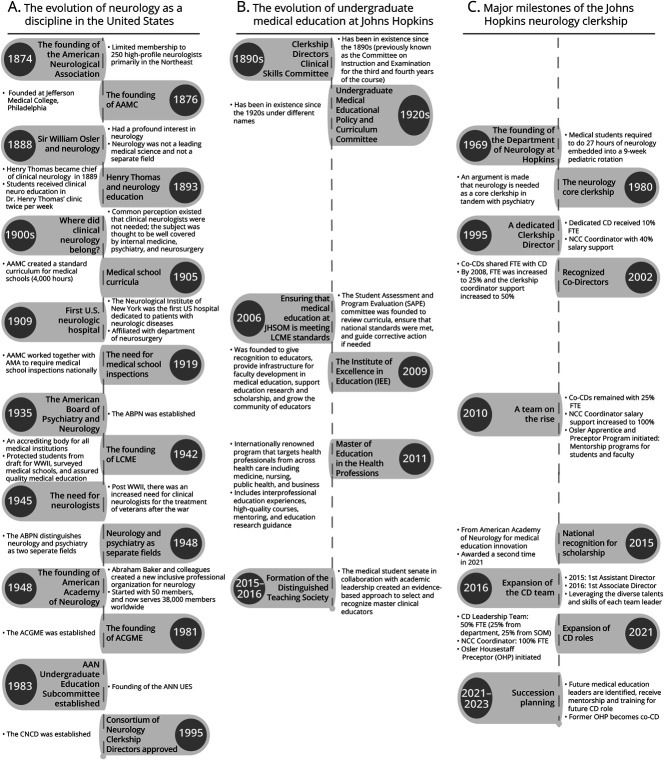
The Evolution of Neurology Education at Johns Hopkins and in the United States (A) The evolution of neurology as a discipline in the United States. (B) The evolution of undergraduate medical education at Johns Hopkins. (C) Major milestones of the Johns Hopkins Neurology Clerkship. AAMC = Association of American Medical Colleges; AAN = American Academy of Neurology; ABPN = American Board of Psychiatry and Neurology; ACGME = Accreditation Council for Graduate Medical Education; CD = clerkship director; FTE = full-time equivalent; JHSOM = Johns Hopkins School of Medicine; LCME = Liaison Committee on Medical Education; Med Ed = medical education; NCC = Neurology Core Clerkship; SAPE = student assessment and program evaluation; UME = undergraduate medical education; WWII = World War II.

During the early 1900s, neurology was not considered among the leading medical sciences in the United States nor was it considered a separate field.^10,11^ The American Neurological Association (ANA), founded in 1874, restricted its exclusive membership to 250 high-profile neurologists (Figure 2A), and the American Academy of Neurology (AAN) was not established until 1948.^12^ Dr. Henry Thomas, president of ANA in 1911, envisioned a Neurological Institute at Johns Hopkins but died before its realization.^10^

The JHSOM Neurology Department was founded in 1969. Before the 1980s, JHSOM medical students received 27 hours of “neurology clinical exposure” in the 9-week pediatric rotation.^13^ A neurologist and his colleague in psychiatry persuaded the curriculum committee to create and link a required neurology and psychiatry clerkship, “pointing out that one without the other was incomplete” (Moses, email communication, March 2023). From 1980 onward, the neurology clerkship became a stand-alone, 4-week block linked to psychiatry to form an 8-week neurology-psychiatry block (Figure 2C). In 2022, the neurology and psychiatry blocks were unlinked for the first time to increase student flexibility in scheduling rotations and electives.

According to the JHSOM registrar, there have been only 14 neurology CDs to date. The registrar's records were not, in fact, accurate; from 1980 to 1994, the registrar's office listed the current department chair as the de facto neurology CD, signifying the lack of formal recognition of the CD outside the department.^13^ During that period, faculty members were designated by the chair to serve as CD. For this reason, we conducted personal communications with former “chair-designated” CDs to gain their insights and perspectives and to document, for the first time, the CD lineage at Hopkins. For more than 40 years, many early CDs described additional faculty playing significant clerkship roles, often without formal recognition or support.

### Expanding the Hopkins Neurology Curriculum

**Figure 3:**
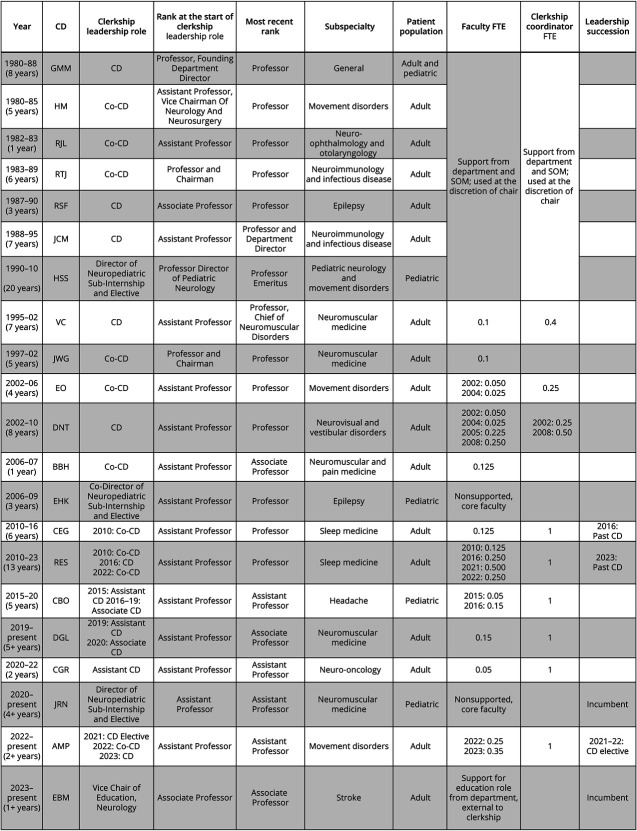
The Evolution of Neurology Clerkship Leaders at Johns Hopkins CD = clerkship director; FTE = full-time equivalent.

In 1995, coincidentally the same year that the AAN Consortium of Neurology Clerkship Directors was created (Persaud, email communication, March 27, 2023), the JHSOM registrar records formally recognized a CD as distinguished from the department director for the first time, granting protected time for education and 10% salary support (Figures 2A and 3).^13,14^ The 1990s saw curricular advancements at JHSOM in the basic and clinical sciences, focusing on case-based learning, localization skills, expanding rotation sites to include some outpatient neurology exposure, and increasing student autonomy.

In 2000, JHSOM appointed its first Vice Dean for Education (Figure 2B). Starting in 2002, a dedicated co-CD role was introduced, with 10% FTE shared between CD and co-CD. Yet the adoption of formal protected time and support at Johns Hopkins did not fully align with the recommended 50% FTE support outlined in the ACE guidelines.^2^

### Increased Recognition and Institutional Support of CD Education and Leadership Innovations

In 2008, JHSOM increased salary support for the CD from 10% to 25% and provided a coordinator with 50% FTE, which became 100% FTE (JHSOM's 50% was matched by 50% from the Neurology Department) in 2010 during a CD transition (Figure 3). The CD used ACE guidelines and AAN recommendations to negotiate the increased support, although not at the recommended 50%.^2^ These changes led to a surge in educational scholarship, curriculum advances, and mentorship programs for students and faculty.^15,16^ The NCC changes coincided with the formation of the Institute of Excellence in Education at Johns Hopkins in 2009 (Figure 2B), supporting faculty development in medical education and scholarship. The NCC used the additional supported time to integrate HSS learning opportunities into the curriculum and to improve the learning environment.

In 2015, the medical student body established the JHSOM Distinguished Teaching Society to formally acknowledge exemplary teachers. In the same year, the NCC director role officially transformed into an NCC leadership team that included a CD, co-CD, coordinator, and assistant director, all receiving formal salary support and role recognition in the JHSOM registrar records. The CD and co-CD shared 25% FTE. JHSOM provided additional FTE support for Pre-Clerkship Exercise (PRECEDE) faculty leads. PRECEDE included clerkship orientation, didactics covering common neurologic topics, case-based learning, localization skills exercises, and lumbar puncture training. PRECEDE FTE was also used to support the assistant director role at 5%. By 2022, with support from the JHSOM, the CDs negotiated an increase in departmental backing so that the total FTE was at the recommended 50%, shared among the current CD and associate CD. The past CD role, described below, received additional PRECEDE support from the JHSOM for the ongoing role as the faculty lead for the clerkship orientation. Since 1989, a neurology pediatric faculty member has played a formal role in the CD leadership team as a pediatric curriculum consultant and host for pediatric neurology subinternship rotations (Figures 2C and 3).

A 2017 national survey reported that among 92 neurology CDs, 71% identified as White, 58% responded as male, 22% identified as Asian, and 12% identified as Latino or Hispanic.^4^ The JHSOM Neurology Department prioritizes a diverse and inclusive leadership team that reflects the evolving demographics of its student body and addresses the gaps identified in the survey.^4^ Since the appointment of the first female CD in 2002 and the first underrepresented in medicine (URiM) directors in 2010, the JHSOM CDs have represented a racially, ethnically, and gender-diverse team. Since 2010, the leadership team has consistently included both a female CD and representation from URiM, non-White individuals, and/or first-generation Americans. This trend mirrors the most recent matriculating class, with over 60% identifying as female, nearly 19% as URiM, and 13% as first-generation students. The JHSOM CDs also have various ranks (from early career faculty to full professor), possess various academic skills (i.e., formal research, medical education, and coaching certifications), and are from several subspecialties with representation from child neurology. This team-based leadership allows well-qualified educators to meet expectations of education scholarship and innovation to provide more rigorous evidence-based standards of curriculum/instructional design (Figure 3).

## Key Lessons Learned From the Past 43 Years

### Building a Team With Diverse Clinical and Academic Strengths

The Johns Hopkins NCC optimized the benefits of a team expansion by establishing a formal leadership team with protected time for their respective roles and institutional salary support (Figure 1B), a trend recognized by ACE. The numerous responsibilities of the CD (i.e., curricular development, education research, and faculty development; Figure 1A)^1^ can be divided among the leadership team members based on their complementary interests, talents, and skillsets. For instance, one CD could focus on mentoring academic scholarship, another on curricular development and faculty support, and another on student and faculty evaluation and feedback. One CD might be adept at discussing constructive feedback with a student, whereas another can efficiently analyze the end-of-clerkship survey data to help guide future decisions. Regularly held collaborative meetings allow for teamwork in addressing curricular innovation, growth, and support of the needs or challenges faced by the learners. In a CD leadership team, the work is shared, there is a community of practice, and each team member can build their education portfolio based on their professional development goals without feeling overwhelmed or risking burnout.^17^ There is a national trend toward increasing support for neurology CDs in the United States and for developing CD teams; yet nearly half of CDs still lack institutional resources to create a leadership team.^4^

### Fostering Medical Education as a Career Path

Embracing a team-based leadership approach can enhance education innovation and cultivate mentorship, with a particular focus on professional development, professional identity formation, and scholarship. This approach aligns with the expanded definition of CD responsibilities (Figure 1, A and B). For example, the Johns Hopkins Osler Apprenticeship (OA) program provides medical students with experiential learning opportunities in medical education: apprentices actively engage in NCC leadership meetings and collaborate on education initiatives. They also contribute to the refinement of the clerkship through their feedback, assist in program building, and engage in education research under the guidance of the CDs.

The OA program built on this success by extending to housestaff, enabling neurology fellows to acquire essential technical, administrative, and teaching skills needed by educators. In addition, the NCC leadership introduced the Osler Preceptors in Neurology program to recognize teaching faculty. These collaborative efforts between students, housestaff, and faculty members create a vibrant community of practice that exchanges experiences and implements evidence-based strategies. Notably, much of the momentum in education scholarship and program development stems from the collaborative efforts of the learners and faculty members for whom it is designed.

### Planning and Supporting CD Succession

A traditional academic model without succession planning leads to stagnation of leadership turnover. It also limits opportunities for advancement into formal roles with protected time and salary support.^18,19^ At JHSOM, CD or co-CD roles have ranged from 1 to 12 years, with a mean of 5.6 years (Figure 3). In 2016 and 2022, past CD and CD elect roles were established, respectively, providing natural transition phases. Formal salary support for both past CD and CD-elect was established in 2022. Past CDs remain connected to the team and often provide mentorship, guidance, historical context, relevant insight, and scholarly contributions. In medical education, a leadership team with succession planning allows for early-career faculty growth and allows experienced CDs to explore new directions. Developing the rising CD and supporting the past CD maximize their strengths and support the institution's mission.^20^ The model of succession planning (Figure 1C) can extend beyond clerkships, benefiting various leadership hand-offs within academia.

## Final Thoughts: A Strong and Diverse Team Allows for Adaptability and Resilience

Despite the increasing burden of neurologic diseases worldwide, there remains a shortage of neurologists, with approximately 3% of graduating medical students matriculating into neurology residencies.^21‐26^ “Neurophobia”—the apprehension learners feel when integrating neuroscience and clinical neurology—is often cited as a challenge to developing interest in neurology.^27‐29^ Neurology educators at JHSOM know that a student's rating of the neurology clerkship correlates with the likelihood of pursuing neurology as a career.^30^ In the past several years, changes to support the growth of the clerkship leadership team have directly correlated with an increase in NCC student satisfaction, as indicated by the Medical School Graduation Questionnaire. The percentage of students rating the clerkship as excellent has risen from 39.2% in 2017 to 63.4% in 2022, surpassing national ratings (43.6%).^31^ Furthermore, the number of medical students applying for adult and pediatric residency programs at JHSOM has doubled in the past decade. In 1987, only 1 medical graduate entered a neurology residency program (Chaudhry, email communication, March 14, 2021). Between 2013 and 2023, the average percentage of graduating medical students matching into neurology residency was 4%, peaking at 6% in 2022.^32^ Although these recent numbers at Hopkins surpass the national average, significant work remains for neurology medical educators worldwide to address the increasing workforce deficit. In the United States alone, the deficit is projected to reach 19% by 2025, highlighting the urgent need to meet the growing demand for neurologic care.^22^

Future perspectives in neurology education may involve expectations of CDs to develop new competencies in HSS and precision education. In other words, CDs could be required to demonstrate experience in areas of medical education that might not have existed during their training. Increasing oversight and use of metrics with accrediting bodies could lead to more centralization of CD administration and teaching experiences. Suggestions to reduce medical school to 3 years will require educators to strategize and advocate for neurology education, considering the low matriculation rate of students into neurology and the persistence of neurophobia. There is a growing focus on developing an evidence-based approach to determine which elements of CD administrative and education structures predict higher numbers of students entering neurology. Factors such as the number of members on the directorial team, salary support, or the rotation's duration could potentially affect the number of students choosing neurology. Further study is required.

Clerkship education—not only in neurology but across disciplines—continues to evolve in educational scholarship, innovation, leadership, professional development, and succession planning while meeting modern education metrics. This retrospective look at the history and evolution of the neurology clerkship from one institution's perspective over the last 43 years can provide the basis for the future: a roadmap for national clerkship education guidelines and institutional adaptation. We encourage other institutions to chronicle their clerkship education history and review national expectations and guidelines to build a roadmap for CDs because they continue curricular advancement, meet the ever-expanding national definition of CD responsibilities, and garner institutional support for the future of andragogical education.

## Appendix Authors

**Table:**

Name	Location	Contribution
Ashley Paul, MD	Department of Neurology, School of Medicine, Johns Hopkins University, Baltimore, MD	Drafting/revision of the manuscript for content, including medical writing for content; major role in the acquisition of data; study concept or design; analysis or interpretation of data
Doris G. Leung, MD, PhD	Department of Neurology, School of Medicine, Johns Hopkins University, Baltimore, MD	Drafting/revision of the manuscript for content, including medical writing for content
Carlos G. Romo, MD	Department of Neurology, School of Medicine, Johns Hopkins University, Baltimore, MD	Drafting/revision of the manuscript for content, including medical writing for content
Vinay Chaudhry, MD	Division of Neuromuscular Disorders, School of Medicine, University of North Carolina, Chapel Hill	Drafting/revision of the manuscript for content, including medical writing for content; major role in the acquisition of data
Justin C. Mcarthur	Department of Neurology, School of Medicine, Johns Hopkins University, Baltimore, MD	Drafting/revision of the manuscript for content, including medical writing for content; major role in the acquisition of data
Eric H. Kossoff, MD	Department of Neurology, School of Medicine, Johns Hopkins University, Baltimore, MD	Major role in the acquisition of data
Jessica Nance, MD, MS	Department of Neurology, School of Medicine, Johns Hopkins University, Baltimore, MD	Major role in the acquisition of data
Charlene E. Gamaldo, MD	Department of Neurology, School of Medicine, Johns Hopkins University, Baltimore, MD	Drafting/revision of the manuscript for content, including medical writing for content; major role in the acquisition of data; study concept or design; analysis or interpretation of data
Rachel Marie E. Salas, MD, MEd	Department of Neurology, School of Medicine, Johns Hopkins University, Baltimore, MD	Drafting/revision of the manuscript for content, including medical writing for content; major role in the acquisition of data; study concept or design; analysis or interpretation of data

## Glossary

AAN: American Academy of Neurology
ACE: Alliance for Clinical Education
ANA: American Neurological Association
CD: clerkship director
FTE: full-time equivalent
HSS: health systems science
JHSOM: Johns Hopkins School of Medicine
NCC: Neurology Core Clerkship
OA: Osler Apprenticeship
PRECEDE: Pre-Clerkship Exercise
URiM: underrepresented in medicine
